# Zooplankton Feeding on the Nuisance Flagellate *Gonyostomum semen*

**DOI:** 10.1371/journal.pone.0062557

**Published:** 2013-05-07

**Authors:** Karin S. L. Johansson, Tobias Vrede, Karen Lebret, Richard K. Johnson

**Affiliations:** 1 Department of Aquatic Sciences and Assessment, Swedish University of Agricultural Sciences, Uppsala, Sweden; 2 Department of Biology, Aquatic Ecology, Lund University, Lund, Sweden; Institute of Marine Research, Norway

## Abstract

The large bloom-forming flagellate *Gonyostomum semen* has been hypothesized to be inedible to naturally occurring zooplankton due to its large cell size and ejection of long slimy threads (trichocysts) induced by physical stimulation. In a grazing experiment using radiolabelled algae and zooplankton collected from lakes with recurring blooms of *G. semen* and lakes that rarely experience blooms, we found that *Eudiaptomus gracilis* and *Holopedium gibberum* fed on *G. semen* at high rates, whereas *Daphnia cristata* and *Ceriodaphnia* spp. did not. Grazing rates of *E. gracilis* were similar between bloom-lakes and lakes with low biomass of *G. semen*, indicating that the ability to feed on *G. semen* was not a result of local adaptation. The high grazing rates of two of the taxa in our experiment imply that some of the nutrients and energy taken up by *G. semen* can be transferred directly to higher trophic levels, although the predominance of small cladocerans during blooms may limit the importance of *G. semen* as a food resource. Based on grazing rates and previous observations on abundances of *E. gracilis* and *H. gibberum*, we conclude that there is a potential for grazer control of *G. semen* and discuss why blooms of *G. semen* still occur.

## Introduction

*Gonyostomum semen* (Ehrenberg) Diesing [Raphidophyceae] is a large flagellate that can form dense blooms in freshwater ecosystems, during which it can contribute more than 95% to the total phytoplankton biomass [1]. Although several marine raphidophyte species may form highly toxic blooms [2], we are not aware of any studies reporting toxin production by *G. semen*. Blooms of *G. semen* can, however, restrict the use of freshwater ecosystems due to its ability to cause skin irritation to humans and clogging of filters for water treatment [3], [4]. Blooms mainly occur in small lakes with high dissolved organic carbon (DOC) content and low pH [5], [6], but have also been reported from other lake types and reservoirs [3], [7], [8]. Several studies have shown an increase in the geographical distribution and bloom incidence of *G. semen* in Northern Europe during recent decades [5], [9], [10]. Increasing levels of DOC in freshwaters and rising temperatures [11], [12] are potential drivers of the expansion, as the growth of *G. semen* is enhanced by humic substances and both growth and recruitment from benthic resting stages are temperature-dependent [5], [13]. Some authors have also suggested that the expansion of *G. semen* could reflect an ongoing invasion process with current colonization of suitable habitats [5].

Fatty acid analyses of seston from lakes dominated by *G. semen* indicate that this alga has a high content of nutritionally valuable fatty acids [14]. However, *G. semen* has several physical adaptations that could limit grazing by zooplankton and limit the transfer of these fatty acids as well as other biochemicals, mineral nutrients and energy to higher trophic levels. The cell size of *G. semen* (length 36–92 µm, width 23–69 µm [13]) is above the preferred size range for many filter-feeding zooplankton (<20 µm for smaller cladocerans and <50 for larger cladocerans [15]). In addition, the fragile cells lack a cell wall and burst upon physical stimulation, releasing slimy threads (trichocysts) with a length up to 200 µm [3]. Feeding experiments using filamentous algae or cellulose fibres have shown that long, filamentous structures can interfere with the filter feeding of cladocerans [16], [17]. Filaments may enter the food groove of cladocerans but are rejected by clearing of the food groove because they cannot be ingested, resulting in energy losses and simultaneous rejection of other food particles [16]. In contrast, calanoid copepods feed selectively and often prefer large phytoplankton. For example, DeMott and Watson [18] showed feeding by calanoid copepods on *Pediastrum* colonies with a diameter of 80 µm. Hence, calanoid copepods could potentially feed on *G. semen*. Nonetheless, Vanderploeg et al. [19] showed an increased handling time and a higher frequency of unsuccessful captures when *Diaptomus sicilis* were feeding on filamentous compared to spherical algae. The copepods were able to facilitate ingestion by manipulating the orientation of the filaments and were also observed biting off parts of them, but many of the captures eventually resulted in the loss of the filament. Feeding efficiency of calanoid copepods could potentially be reduced in a similar manner by the numerous trichocysts ejected by *G. semen*.

Previous feeding experiments with *G. semen* have shown contrasting results. Lebret et al. [20] observed significant grazing on *G. semen* by *Daphnia magna*, but neither *D. pulex* nor the calanoid copepod *Eudiaptomus gracilis* had any significant effect on *G. semen* cell density. Williamson et al. [21], on the other hand, observed a significant reduction in the number of *G. semen* cells in treatments with the calanoid copepod *Diaptomus oregonensis* and estimated that 44% of their diet consisted of *G. semen*. They also recorded some feeding on *G. semen* by *Daphnia pulicaria* (estimated to 27% of diet). Both of these studies were, however, based on cell counts and it is thus not clear whether the animals actually ingested *G. semen* or if the reduction in cell numbers were due to mortality caused by damage to the cells.

The aim of our study was to investigate if zooplankton species naturally occurring in Swedish brown-water lakes are able to ingest *G. semen* and answer the questions 1) whether *G. semen* is a potential food source for zooplankton in boreal lakes and 2) whether lack of grazers able to feed on *G. semen* could explain the occurrence of mass developments of this alga. We hypothesized that calanoid copepods would be able to feed on *G. semen* to some extent, but that the small cladocerans occurring during late summer, when *G. semen* blooms normally peak, would not be able to ingest the alga. We used a radiolabelling method, allowing us to make quantitative estimates of ingestion rates and avoid confounding effects of algal cells that were damaged but not ingested.

## Materials and Methods

### Algal Cultures

Laboratory cultures of *G. semen* and the small chlorophyte *Pseudokirchneriella subcapitata* (Korshikov) Hindák were used in our experiment. *P. subcapitata* was expected to be eaten by all zooplankton taxa in the experiment and was used as a control for assessing the condition of the experimental animals. The *G. semen* culture (GSLI20) originated from Liasjön in southern Sweden and the culture of *P. subcapitata* (NIVA-CL1) came from the culture collection at NIVA (Norwegian Institute for Water Research, Oslo). The algal cultures used were non-axenic single-cell cultures grown in modified Wright’s cryptophyte medium (MWC) with extra Se (1.2 µg L^−1^, final concentration) [22]. Algae were cultured at 20°C under a 14∶10 light:dark cycle and a light intensity of 20 µmol m^−2^ s^−1^. Cultures were labelled with 7.4 kBq mL^−1^ of H^14^CO_3_ (PerkinElmer, NEC086H005MC) for 28–30 h. The average cell volume of *G. semen* was 25 000 µm^3^ (length 62, width 35 µm) and the average cell volume of *P. subcapitata* was 96 µm^3^ (length 7, width 2 µm).

### Zooplankton

Zooplankton were collected from four lakes in southern Sweden, two of which have recurring blooms of *G. semen* (Bäen and Älgarydssjön) and two in which *G. semen* occurs but usually does not bloom (Hagasjön and St Skärsjön). The lakes were all small, humic forest-lakes with similar trophic states (Table 1). Zooplankton were collected by vertical net hauls in the pelagic zone and transported back to the lab in 5 L of lake water filtered through a 45 µm mesh. The animals were kept dark and cool in a cooling box during transportation and upon arrival they were stored overnight in darkness at 15°C. The next day, at room temperature (∼20°C), a pipette was used to transfer 10–15 animals of each taxon to beakers with GF/C-filtered lake water. The taxa used in the experiment were the most abundant crustaceans collected from the lakes, i.e. *Eudiaptomus gracilis* Sars (adult males and females), *Daphnia cristata* Sars, *Diaphanosoma brachyurum* Liévin, and *Ceriodaphnia* spp. *Holopedium gibberum* Zaddach was present in low numbers in one of the lakes and individuals sufficient for one incubation with *G. semen* were isolated. Two to three taxa were added to the same beaker. Average body lengths of experimental animals are given in Table 2. According to Swedish law, no permits are required for field sampling of plankton. No ethical permits were required for this study and no protected organisms were sampled.

**Table 1:** Lake characteristics.

	Bäen	Älgarydssjön	Hagasjön	St Skärsjön
Latitude (°N)	56.245	57.176	57.337	56.675
Longitude (°E)	14.378	14.273	13.714	13.071
Surface area (km^2^)	0.50	0.33	0.12	0.30
Mean depth (m)	3.4	1.4	3.7	3.9
Secchi depth (m)	1.9±0.50	1.1±0.15	2.2±0.53	3.1±0.62
pH	5.9±0.11	5.4±0.27	6.5±0.16	7.0±0.16
Tot-N (µg L^−1^)	610±160	610±110	420±42	350±92
Tot-P (µg L^−1^)	12±0.58	21±4.4	8.3±1.5	7.9±1.6

Location and physico-chemical characteristics of the four lakes from which zooplankton were collected. Secchi depth and water chemistry values are growing season (April-October) averages ± standard deviation from 2011. Data is available at www.slu.se/aquatic-sciences.

**Table 2:** Experimental animals.

	Body length (average length ± SD, mm)			
	Bäen	Hagasjön	St Skärsjön	Älgarydssjön
*Eudiaptomus gracilis*	1.07±0.07	1.05±0.07	1.01±0.08	1.05±0.08
*Diaphanosoma brachyurum*	0.77±0.08	0.73±0.10	0.72±0.10	
*Daphnia cristata*		0.72±0.09	0.63±0.09	
*Ceriodaphnia* spp.	0.47±0.05			0.44±0.04
*Holopedium gibberum*				Not measured

Zooplankton taxa included in the experiment with average body length of animals collected from the four different lakes.

### Experimental Conditions

At the start of the experiment, labelled algae were added to the beakers to a total volume of 80 mL and a final density of 2 mgC L^−1^ (730 cells mL^−1^) in *G. semen* treatments (assuming 11% C of fresh weight, [23]) and 18 mgC L^−1^ (1 160 000 cells mL^−1^) in *P. subcapitata* treatments (assuming 16% C of fresh weight, [23]). The difference in cell densities was due to a calculation error, but we consider this difference to be of minor importance since *P. subcapitata* incubations were only used to assess the physiological condition of the experimental animals and enable us to rule out effects of poor health on the ingestion of *G. semen*.

The experiment was conducted in a water bath at 20°C and was terminated after 10 min (shorter than gut passage time for *Daphnia* [24]), when the animals were collected on nylon mesh mounted on a Plexiglas cylinder, rinsed at least three times in carbonated water to kill the animals and remove any labelled food adhering to the outside of their bodies, and frozen at −20°C. Incubations with radiolabelled algae were done in triplicates for all taxa except for *Diaphanosoma brachyurum* from Bäen, for which incubations were done in duplicate, and *Holopedium gibberum* from Älgarydssjön for which we could only isolate enough animals for one incubation with *G. semen*. An additional replicate of each taxon was killed and frozen before the experiment for use as blanks. One sample (2 mL) of unlabeled algal culture and one of labelled algae was collected on polycarbonate filters (1 µm, MSI, Westboro), which were transferred to glass scintillation vials (PerkinElmer, 6000134) and frozen at −20°C.

### Scintillation Counting

Animals were freeze-dried, measured under a dissecting microscope and transferred to glass scintillation vials. *D. cristata*, *Ceriodaphnia* spp. and *Diaphanosoma* were measured from the top of the head to the base of the spine or mucro, *E. gracilis* were measured from the top of the head to the end of the furcal rami, and *H. gibberum* were not measured. 6–10 animals per taxon were retrieved from each replicate. Zooplankton and filters with algae were dissolved for 4 h in 2 mL tissue solubilizer (Soluene 350, PerkinElmer, 6003038) at 60°C. After cooling to room temperature, 10 mL of scintillation cocktail (Ultima Gold, PerkinElmer, 6013321) was added to the samples and they were incubated in the dark at room temperature for 24 h. The samples were then counted in a scintillation counter (Beckman LS 6000TA) up to 40 000 counts or for a maximum of 30 min.

### Data Analysis

Ingestion rates (cells ind^−1^ min^−1^) were calculated by dividing blank-corrected specific activities in animals by the blank-corrected specific activity per algal cell and the number of zooplankton individuals per sample. Differences in ingestion rates between taxa and lakes (combined) were tested using one way ANOVA (analysis of variance) and Tukey’s HSD (honestly significant difference) test for post hoc comparisons on log-transformed data in JMP version 10 (SAS Institute Inc.). The results were not corrected for size-specific differences in feeding rates.

## Results

**Figure 1:**
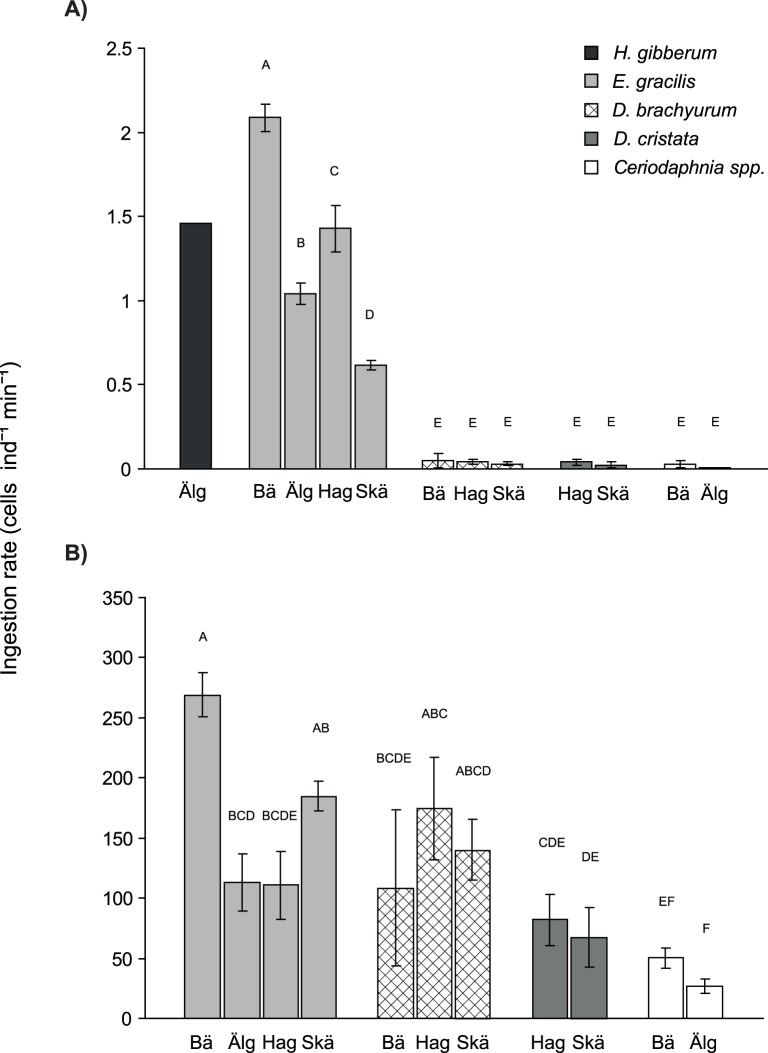
Ingestion rates. Ingestion rates (cells ind^−1^ min^−1^) of zooplankton from the four different lakes feeding on A) *G. semen* and B) *P. subcapitata*. Lakes Bäen and Älgarydssjön have a history of recurring *G. semen* blooms whereas Hagasjön and St Skärsjön usually have low biomasses of *G. semen*. Bars not connected by the same letter are significantly different from each other (one way ANOVA with Tukey’s HSD comparisons, p<0.05). The *H. gibberum* sample comprised only one replicate and was not included in the statistical analysis. Error bars represent standard deviation.

The calanoid copepod *E. gracilis* fed on *G. semen* with an average ingestion rate of 1.3 cells ind^−1^ min^−1^ (Fig. 1A), corresponding to a daily (24 h) ingestion of 0.047 mm^3^ ind^−1^ d^−1^. In the one treatment containing *H. gibberum*, this species showed a similar ingestion rate to the copepods (1.5 cells ind^−1^ min^−1^, Fig. 1A, corresponding to 0.053 mm^3^ ind^−1^ d^−1^). By contrast, the average ingestion rates of *D. brachyurum*, *D. cristata*, and *Ceriodaphnia* spp. were 0.041, 0.032, and 0.018 cells ind^−1^ min^−1^, respectively (Fig. 1A). These ingestion rates correspond to 0.0015, 0.0012, and 0.00066 mm^3^ ind^−1^ d^−1^. Possibly, the radioactive signal in small cladocerans reflects feeding on parts of broken *G. semen* cells and/or bacteria that had been labelled by consumption of algal exudates.

One way ANOVA showed significant differences between taxa and also between lakes for animals incubated with *G. semen* (p<0.0001, *r^2^* = 0.997, *F*_10,21_ = 756, *H. gibberum* not included, Fig. 1A). Tukey’s HSD test showed that the ingestion rates of *E. gracilis* on *G. semen* were significantly higher than those of the cladocerans *D. brachyurum*, *D. cristata*, and *Ceriodaphnia* spp. (Fig. 1A). The pattern for copepods vs. small cladocerans was repeated across lakes, i.e. copepods from one lake always had a higher ingestion rate than cladocerans from the same lake even though the feeding rates varied between lakes. The pattern of among-lake differences was not related to the bloom history of the lakes (Fig. 1A).

All zooplankton taxa incubated with the control alga *P. subcapitata* grazed on this species. *E. gracilis* and *D. brachyurum* showed the highest ingestion rates on *P. subcapitata* (Fig. 1B). The average ingestion rate of *E. gracilis* was 170 cells ind^−1^ min^−1^ (Fig. 1B), corresponding to 0.023 mm^3^ ind^−1^ d^−1^, and the average ingestion rate of *D. brachyurum* was 150 cells ind^−1^ min^−1^ (Fig. 1B), corresponding to 0.020 mm^3^ ind^−1^ d^−1^. Ingestion rates of *D. cristata* and *Ceriodaphnia* spp. were lower – on average 75 cells ind^−1^ min^−1^ (Fig. 1B), corresponding to 0.010 mm^3^ ind^−1^ d^−1^, for *D. cristata* and 39 cells ind^−1^ min^−1^ (Fig. 1B), corresponding to 0.0053 mm^3^ ind^−1^ d^−1^. One way ANOVA showed significant differences between taxa and lakes (*p*<0.0001, *r^2^* = 0.947 *F*_10,21_ = 17.6, Fig. 1B).

## Discussion

The high ingestion rates of *E. gracilis* and *H. gibberum* incubated with *G. semen* in our experiment show that some naturally occurring zooplankton in boreal humic lakes can feed on this bloom-forming alga. The daily (24 h) ingestion rates of *E. gracilis* feeding on *G. semen* (0.053 mm^3^ ind^−1^ d^−1^) were almost twice as high as the maximum daily ingestion rate of *E. gracilis* on the readily ingested *Scenedesmus quadricauda* reported by Horn (0.030 mm^3^ ind^−1^ d^−1^ [25]) and more than twice as high as the ingestion rates on *P. subcapitata* in our experiment (0.023 mm^3^ ind^−1^ d^−1^). This implies the possibility of a direct transfer of energy and nutrients from *G. semen* to higher trophic levels in pelagic food webs, despite the physical defences of *G. semen*. However, a previous field study [26] of zooplankton assemblages in eight boreal, humic lakes showed predominance of small cladocerans in late summer, with *Ceriodaphnia* spp. predominating in lakes with recurring *G. semen* blooms and *Daphnia cristata* in lakes without blooms. Since none of the small cladocerans used in our experiment (*Ceriodaphnia* spp., *Daphnia cristata* and *Diaphanosoma brachyurum*) fed on *G. semen*, the trophic transfer from phytoplankton is likely reduced in bloom-lakes compared to other humic lakes with a larger proportion of small edible phytoplankton taxa [26], [27]. Instead, there may be a shift to an increased importance of microbial pathways during *G. semen* blooms, as indicated by a large proportion of bacterial fatty acid markers in *Ceriodaphnia* spp. collected from lakes with *G. semen* blooms (Johansson, unpublished data).

The results of our experiment contradict those of Lebret et al. [20], who found that *E. gracilis* did not feed on *G. semen* and hypothesized that the lack of any naturally occurring zooplankton able to feed on *G. semen* could explain the formation of blooms. The copepods used in the experiment by Lebret et al. [20] originated from a lake without *G. semen*, which could indicate that they were not adapted to feeding on this alga. However, our results do not support this conjecture, since copepods collected from both lakes with recurring blooms and lakes that usually have low quantities of *G. semen* were feeding on *G. semen*. As Lebret et al. [20] did not observe grazing by copepods on either *G. semen* or *P. subcapitata,* whilst our experiment showed high ingestion rates on both algae, poor condition of the copepods in their experiment may explain the difference between the two studies.

In a previous field study including four lakes with recurring *G. semen* blooms, we found that pre-bloom (May) abundances of calanoid copepods were on average 3.2 individuals L^−1^ and the abundance of *H. gibberum* in the two lakes where this taxon occurred was on average 9.1 individuals L^−1^ (Johansson et al., unpublished data). Using the average ingestion rates from our experiment and observed field densities results in a daily (24 h) consumption of approximately 6 000 *G. semen* cells L^−1^ d^−1^ by copepods and 20 000 cells L^−1^ d^−1^ by *H. gibberum*. Hence, considering the poor growth and slow recruitment of *G. semen* from resting stages at low temperatures [5] it seems plausible that *E. gracilis* and/or *H. gibberum* could be able to control the abundance of *G. semen* during early summer. However, field observations do not support the contention that zooplankton are able to prevent the development of *G. semen* blooms.

Laboratory feeding experiments in which animals are incubated with a dense monoculture of algae may overestimate feeding on the tested algal species in natural phytoplankton assemblages, since the algal species of interest often occurs at lower abundance, in a more patchy distribution and mixed with other food particles. Hence, low abundance of *G. semen* and presence of other food resources during the pre-bloom period may reduce the feeding of *E. gracilis* and *H. gibberum* on *G. semen*. The amount of *G. semen* in our experiment does, however, correspond to natural abundances recorded during intense blooms (Johansson et al., unpublished data) when *G. semen* almost completely dominates the phytoplankton assemblage, suggesting that grazing on *G. semen* may be substantial during the bloom period.

Keeping the experimental animals in filtered lake water prior to the incubations may have resulted in starvation and overestimation of feeding rates in our experiment [28]. If the results of Lampert et al. [28] are applicable to other cladoceran taxa than *Daphnia*, we may have overestimated the ingestion rate of *H. gibberum* feeding on *G. semen* by 30–50% compared to pre-fed animals. *E. gracilis* has, however, been shown to be relatively tolerant to starvation [29]. In addition, since Lampert et al. [28] showed that the duration of the starvation period affected ingestion rates, we should have observed a large variation between the replicates in our experiment, as they were isolated in random order and consequently starved for between <1 h and ca 10 h. The variation between replicates was, however, small, implying that starvation had a minor influence on the feeding rates of *E. gracilis* on *G. semen*.

Late summer declines of *H. gibberum* populations have been observed by several authors [30]–[32] suggesting a decreased grazing pressure from this zooplankton species in late summer. In addition, declining *H. gibberum* abundance may also have an indirect positive effect on *G. semen* densities, since the recruitment of *G. semen* from benthic resting stages has been shown to be reduced in the presence of grazers [33]. Differences in the vertical distribution of *G. semen* and zooplankton populations may also limit grazing on *G. semen*. The nighttime migration of *G. semen* to the hypolimnion likely protects the alga from zooplankton grazing during the dark hours, since most zooplankton do not enter this cold and often anoxic stratum [31]. Since the daytime vertical position of *G. semen* appears to be regulated by light conditions [31], the relative vertical positioning of zooplankton and *G. semen* may also vary during the day, between days with different light intensities and between lakes with different transparencies. Hence, daily grazing rates on *G. semen* likely vary temporally within the same lake and may even differ between lakes with similar zooplankton assemblages, making predictions of actual grazing rates on *G. semen* a challenge.

Our observations of high grazing rates on *G. semen* by both *E. gracilis* and *H. gibberum* support the use of biomanipulation as a means of controlling *G. semen*. Manipulation of zooplankton by fish removal has, to our knowledge, only been used once to control *G. semen* blooms. A recent study of biomanipulation of algal blooms through selective removal of zooplanktivorous fish included three lakes with recurring blooms of *G. semen* [34], but an increase in zooplankton biomass was unfortunately only achieved in one lake. In this lake, there was a reduction in *G. semen* biomass in the years following manipulation, possibly due to the increase in biomass of calanoid copepods. To further our understanding of the interactions between zooplankton and *G. semen* and enable efficient design of management efforts, future studies should focus on the relative vertical positioning of zooplankton and *G. semen*, grazing rates of diaptomid copepods on *G. semen* when other food sources are available and the drivers of the late-summer decline in *H. gibberum* populations.
